# Prognostic significance of the urokinase plasminogen activator system in tissue and serum of dogs with appendicular osteosarcoma

**DOI:** 10.1371/journal.pone.0273811

**Published:** 2022-09-29

**Authors:** Arata Matsuyama, Geoffrey A. Wood, Rachael Speare, Courtney R. Schott, Anthony J. Mutsaers

**Affiliations:** 1 Department of Biomedical Sciences, Ontario Veterinary College, University of Guelph, Guelph, Canada; 2 Department of Pathobiology, Ontario Veterinary College, University of Guelph, Guelph, Canada; 3 Department of Clinical Studies, Ontario Veterinary College, University of Guelph, Guelph, Canada; Colorado State University, UNITED STATES

## Abstract

Urokinase plasminogen activator (uPA) and its receptor uPAR promote cancer invasion and metastasis and are emerging therapeutic targets in both human and canine malignancies. While their clinical significance is well-characterized in multiple human tumor types, studies investigating their roles in osteosarcoma are lacking. The objectives of this study were to characterize serum and tissue uPA/uPAR expression in dogs with osteosarcoma and assess the prognostic significance. Serum samples and a tissue microarray of canine appendicular osteosarcoma were analyzed for uPA and uPAR expression by ELISA (n = 49) and immunohistochemistry (n = 38), respectively. Serum uPA activity was also measured by a chromogenic assay (n = 25). Survival analysis was performed by Kaplan-Meier survival analysis, log rank test, and Cox regression analysis. Serum uPA level was significantly higher in dogs with osteosarcoma than clinically healthy control dogs (median 1905 vs 1440 pg/ml, p = 0.008). The majority of canine osteosarcoma tissues expressed uPA (75.9%) or uPAR (77.6%), with 70.7% dual-positivity, indicating autocrine/paracrine activation of the pathway. Survival analysis revealed shorter progression free survival (PFS) in dogs with high serum uPA level in a discovery cohort (n = 29; median PFS 94 vs 266 days, p = 0.003) but not in a validation cohort (n = 23; median PFS 167 vs 490 days, p = 0.16). The difference was significant when both cohorts were combined (n = 49; median PFS 128 vs 266 days, p = 0.003). Serum uPAR and tissue uPA/uPAR levels were not prognostic. In Cox multivariate analysis, high serum uPA level and activity were both associated with poor prognosis, independent of serum ALP, tumor location, and peripheral lymphocyte/monocyte counts. These results indicate high utilization of the uPA pathway and association with disease progression in canine osteosarcoma. Further study involving prospective evaluation to confirm the prognostic significance is warranted. The high prevalence of tissue uPA and uPAR expression suggests the uPA system as a potential therapeutic target in canine osteosarcoma.

## Introduction

Appendicular osteosarcoma is the most common and an aggressive bone cancer in dogs, with up to 90% of affected dogs developing metastasis following standard of care [1]. While the addition of systemic chemotherapy post limb amputation can extend survival from 3–5 to 8–11 months, the majority of affected dogs eventually succumb to distant metastasis [2–7]. Despite intense interest in developing better treatments for osteosarcoma metastasis, the outcome has remained static for decades. Identifying key molecules involved in the metastatic cascade in osteosarcoma may help to find a successful therapy to help control this aggressive neoplasia.

Urokinase plasminogen activator (uPA) and its receptor uPAR play critical roles in cancer metastasis, invasion, and angiogenesis [8]. uPA is a trypsin-like serine protease, which facilitates cell proliferation and migration following activation of plasminogen to plasmin and secondary proteolysis of growth factors and the extracellular matrix [8]. Activation of uPAR following uPA binding also induces cell growth, invasion, and increased cell survival through interaction with over 30 receptors such as integrins and epidermal growth factor receptor (EGFR) with subsequent activation of their intracellular signaling pathways [8, 9]. Furthermore, pre-uPA and uPA/uPAR complex bind to nucleolin, a nucleocytoplasmic transporter protein, and translocate to the nucleus, where they regulate transcription of genes that promote angiogenesis [10–12].

Expression of uPA and uPAR in human osteosarcoma has been reported, suggesting the probable contribution of the uPA system to its malignant behavior [13]. uPA and uPAR expression by osteosarcoma is increased locally at the invasive front, enhancing its stromal degradation and local invasion [14]. Notably, an association between enhanced metastatic behavior and high uPA excretion as well as uPAR membranous expression was shown in a proteomic *in vitro* study of human osteosarcoma, emphasizing its key role in metastasis development [15]. While these findings were predominantly reported in the human and the mouse, tissue uPAR gene expression has also been confirmed previously in canine osteosarcoma through the RNA sequencing of 31 clinical specimens [16].

In regard to the cumulating evidence of uPA and uPAR expression in a wide variety of cancers including osteosarcoma, the uPA system has been a candidate for patient prognostication both via tissue analysis and liquid biopsy in multiple human cancers [17–23]. uPAR is an intracellular domain-free receptor and is released from cellular the membrane as a soluble receptor (suPAR) when its glycosylphosphatidylinositol anchor is cleaved [24]. Subsequently both patient uPA and suPAR expression status can be readily assessed using blood or urine samples without requiring tissue biopsy. Indeed, circulating suPAR level can less-invasively estimate the tissue uPAR expression level, as their significant correlation was shown in a cohort of 77 human patients with soft tissue sarcoma [17].

Given the aggressive behavior of canine osteosarcoma, the biological role of the uPA system in human osteosarcoma’s aggressiveness, and the established prognostic utility of the uPA system in various cancers, we investigated the potential of the uPA system to serve as a prognostic marker in canine osteosarcoma. Use of a circulating biomarker may help to predict prognosis less invasively prior to aggressive therapy and monitor disease progression. While some negative prognostic factors such as humeral and scapular tumor location, increased serum ALP, higher tumor grade, and higher peripheral lymphocyte and monocyte counts have been reported in dogs with osteosarcoma, the clinical use of these factors are limited either as a pre-surgical baseline assessment or due to low reproducibility among publications [25–27]. Therefore, the objectives of the current study were to assess serum and tissue uPA and uPAR expression in dogs with naturally occurring appendicular osteosarcoma and analyze the prognostic significance in cohorts of dogs that underwent a uniform standard of care (SOC) therapy. We hypothesized that dogs diagnosed with osteosarcoma have higher serum uPA and suPAR levels than healthy dogs, and that high tissue and serum levels are associated with shorter survival.

## Materials and methods

### Serum samples

Serum samples from 49 dogs with a histological diagnosis of appendicular osteosarcoma and 6 clinically healthy dogs were used for circulating biomarker assessments. All patient samples were collected before limb amputation as a component of other clinical trials at the Ontario Veterinary College Animal Cancer Centre with the owners’ consent under the animal use ethical approval for the other trials (ICCI-AUP#3603). The clinical samples were divided into two cohorts: discovery and validation. The discovery cohort included 26 samples collected between June 2015 and January 2017. For this discovery cohort, post-amputation samples were also collected immediately prior to the first carboplatin chemotherapy (n = 25). The sample size was based on the number of available previously banked samples, hence no pre-study power analysis was performed. The validation cohort samples were obtained prior to limb amputation between January 2017 and July 2019 from 23 dogs and were subsequently analyzed owing to the prognostic significance of baseline serum uPA level observed in the discovery cohort. Only dogs that had no detectable metastatic disease at diagnosis and that underwent SOC were included. The clinically healthy dogs underwent physical examination, complete blood count, and serum biochemistry to confirm absence of disease and were followed at least for 1 year to confirm a lack of cancer development.

### Serum uPA and suPAR quantification

Commercially available uPA and uPAR ELISA kits (MBS2603084 and MBS737026, MyBioSource Inc., San Diego, CA, USA) were used to quantify serum uPA and suPAR levels in both discovery and validation cohorts according to the manufacturer’s instructions. All serum samples were diluted at 1:4 (uPA) or 1:8 (suPAR) to fit in their standard curves due to their high serum concentration. Absorbance was read by a spectrophotometer at 450 nm and normalized to blank control wells. The serum concentrations were calculated based on the standard curves using either a linear equation for uPA or a four-parameter logistic curve for uPAR. Each test was performed in technical duplicate in the same run and the mean values were used for survival analysis.

### Serum uPA activity

A commercially available uPA chromogenic activity kit (ab108916, Abcam plc, Cambridge, UK) was used to quantify serum uPA activity in the discovery cohort according to the manufacturer’s instructions. After the final incubation, absorbance was read every 30 minutes for 2 hours by a spectrophotometer at 405 nm. Data obtained after a 120-minute incubation was used for analysis. The absorbance was normalized to blank control wells and serum uPA activity was calculated based on a linear equation of the uPA standards. Each test was performed in technical duplicate and the mean values were used for survival analysis.

### Tumor samples

A previously constructed canine osteosarcoma tissue microarray (TMA) at the Ontario Veterinary College was used for immunohistochemical uPA and uPAR expression analysis [28]. Three TMA blocks comprising 327 cores were sectioned at 4 μm and mounted on slides for immunohistochemistry. The TMA contains the osteosarcoma tumors of 55 dogs representing 51 primary appendicular and 27 metastatic sites each with 3 to 6 core replicates distributed across three blocks. The replicate cores originated from different areas of the same tumor specimen to account for intra-tumoral heterogeneity. The primary tumors were collected from surgical biopsies at amputation. The metastatic tumors included 2 metastatic regional lymph nodes sampled at the time of amputation and 25 sites collected at necropsy from 9 dogs. The postmortem metastatic samples included 9 lungs, 4 appendicular bones, 3 lymph nodes, 2 ribs, and one each of liver, mediastinum, muscle, pericardium, pleura, skin, and vertebra. Patient-matched samples both from primary limb amputation and postmortem metastases were available in 5 dogs. Among the 48 dogs with the primary appendicular tumor tissues available, 42 dogs underwent SOC including 21 dogs that were included in the serum discovery cohort.

### Immunohistochemistry

Sections of the canine osteosarcoma TMA were deparaffinized with xylene followed by rehydration with isopropanol. Endogenous peroxidase activity was blocked in 3% hydrogen peroxide for 10 minutes. No antigen retrieval was required. Sections were blocked for 1 hour at room temperature with 5% normal goat serum (Vector Laboratories, Burlington, ON, Canada) in a humidified chamber, then incubated with primary antibodies overnight at 4°C with the following dilution: rabbit anti-uPA polyclonal antibody at 1:100 (NBP1-80632; Novus Biologicals, Littleton, CO, USA) and rabbit anti-uPAR polyclonal antibody at 1:200 (PA5-70606; ThermoFisher, Burlington, ON, Canada). After primary antibody incubation, slides were incubated with biotin-conjugated anti-rabbit IgG secondary antibody (Jackson ImmunoResearch Laboratories, West Grove, PA, USA) for 1 hour at room temperature, followed by Peroxidase-conjugated Streptavidin (Jackson ImmunoResearch Laboratories) and 2,2’-diaminobenzidine substrate (Vector Laboratories). Slides were counterstained with hematoxylin, then immersed in acid alcohol followed by ammonia water. Dehydration was performed with isopropanol followed by xylene. Coverslips were mounted using Richard-Allan Scientific Cytoseal XXL mounting media (Thermo Fisher Scientific, Waltham, MA, United States). All TMA immunohistochemistry was performed in a single experiment including both positive and negative controls. Primary antibody omission was used as a technical negative control, and normal canine cerebellum and canine hemangiosarcoma tissues were used as biological negative and positive controls, respectively. Reactivity of the antibodies in canine tissues was demonstrated by immunoblotting and visualizing bands of the appropriate molecular weight exclusively in positive control tissue.

TMA slides were scanned at 40x resolution using Aperio AT2 brightfield scanner (Leica, Bannockburn, IL, USA) and the images were viewed with Aperio ImageScope software (Leica). The images were assessed by three independent observers (AM, GAW, RS) blinded to clinical, pathological, and TMA parameters. After the initial image review, the following scoring scheme was applied for both uPA and uPAR expression in neoplastic cells: cytoplasmic labeling intensity was assigned 0, 1+, or 2+ and nuclear labeling was either negative or positive. Only cores with cytoplasmic or nuclear positivity in greater than 30% of the osteosarcoma cells were scored 1+/2+ or positive, respectively. The cytoplasm of osteoclasts and vascular endothelial cells were commonly both uPA and uPAR positive, hence these cells were frequently used as a reference within the same cores for cytoplasmic intensity scoring. Cores with low quality or cellularity due to sectioning or processing were excluded. The images were reviewed individually and a consensus was achieved by image re-review and discussion in cases of discrepancy. Among replicate cores from the same tumor, the highest score was used to classify the tumor for further analysis.

After review of all tissue scores, demineralization was found to have significantly increased both uPA and uPAR immunoreactivity, and therefore, only samples not exposed to demineralization solution were included in the final analysis., including 34 primary appendicular and 24 metastatic osteosarcoma from 38 dogs. Among the 34 dogs with the primary appendicular tumor tissues available, 28 dogs underwent SOC, of which 13 dogs were also included in the serum discovery cohort.

### Clinical data

The medical records were reviewed and referring veterinarians and/or clients were contacted for patient follow-up information at the time of study data collection. The data collected include age, sex and body weight upon initial presentation, tumor location, peripheral lymphocyte and monocyte count on complete blood counts and serum ALP result (elevated or normal) obtained presurgically, date of limb amputation, treatment details, results of follow-up staging tests, and date and cause of death or euthanasia. The SOC for dogs with appendicular osteosarcoma at the Ontario Veterinary College during the period of sample collection was limb amputation followed by adjuvant intravenous carboplatin (300 mg/m^2^) every 3 weeks for 4 doses, beginning 10 to 14 days post-operatively. Thoracic radiographs were obtained at the time of diagnosis and at either the third or fourth carboplatin dose, then recommended to continue every 2–3 months thereafter.

### Immunofluorescence

Three osteosarcoma cell lines D17, Dharma, and HOS were used for uPA and uPAR immunofluorescence. D17 was derived from a metastatic canine pulmonary osteosarcoma and was purchased from Sigma-Aldrich/European Collection of Cell Cultures, while Dharma was a previously isolated and validated canine osteosarcoma cell line by one of the authors (AJM) [29]. HOS is a human osteosarcoma cell line purchased from American Type Culture Collection (CRL-1543™; Manassas, VA, USA).

Cells were seeded at 2 x 10^5^ cells/well on 22 mm glass coverslips (Thermo Fisher Scientific, Waltham, MA, USA) inside 12-well plates and incubated for 24 hours. Cells were washed with phosphate buffered saline (PBS) then fixed with 4% paraformaldehyde (Electron Microscopy Sciences, Hatfield, PA, USA) for 10 minutes, followed by permeabilization with 0.1% Triton-X for 10 minutes and subsequent blocking with 5% bovine serum albumin in PBS for 30 minutes. Cells were then incubated with rabbit anti-uPA polyclonal antibody (NBP1-80632; Novus Biologicals) at 1:100 or rabbit anti-uPAR polyclonal antibody (PA5-70606; ThermoFisher) at 1:50 overnight at 4°C on parafilm placed in a humidified chamber. Secondary antibody (Alexa-488-conjugated donkey anti-rabbit IgG) was applied for 1 hour protected from light. Nuclei were stained with 40,6-diamidino-2-phenylindole dilactate for 5 minutes then coverslips were mounted using fluorescent DAKO mounting medium (Agilent Technologies, Santa Clara, CA, USA). Cytoplasmic and nuclear expression was evaluated with Leica DMLB fluorescent microscope (Leica) fitted with a Q imaging QICAM fast 1394 digital camera.

### Statistical analysis

Descriptive statistics were used for continuous data with median values and categorical data with frequencies. For comparisons between pre-/post-amputation and healthy dog samples, the data was first analyzed for normality by the Shapiro-Wilk test. While the differences were compared with either the 2-sample t-test or Wilcoxon rank-sum test between the pre-amputation osteosarcoma and healthy dog samples, paired t-test or Wilcoxon signed-rank test were used for comparison between pre-/post-amputation samples, depending on the normality of the data. Both serum uPA quantity and uPA activity were normally distributed and bivariate correlation analysis was performed by a Pearson’s correlation coefficient. Assessment of relationship between tissue uPA and uPAR expression or between tumor origin (primary vs metastasis) and uPA/uPAR expression were performed by McNemar’s test.

The Kaplan-Meier method was used to calculate median progression free survival (PFS) and overall survival (OS). The definition of PFS was the time elapsed between surgery and first evidence of tumor progression, euthanasia or death. Dogs that were still alive without disease progression at the time of study end were censored from PFS analysis. The definition of OS was the time elapsed between surgery and death or euthanasia owing to any cause. Dogs that were still alive at the end of the study were censored from OS analysis. For survival analysis, dogs with analyzed serum samples were allocated into two groups (low or high) based on cut-off values of serum uPA or suPAR generated by X-Tile 3.5.0 software [30]. For immunohistochemistry scoring data, dogs were grouped into two (negative or positive) or three (negative, 1+ or 2+). The log rank test was used to investigate survival distribution between the groups.

Prognostic variables were also assessed for their effects on survival using a Cox proportional hazards model. Variables assessed include age (continuous), tumor location (categorical; humerus vs others), serum ALP status (categorical; normal vs high), peripheral lymphocyte and monocyte counts (categorical; lower vs higher than median), serum uPA and suPAR level (categorical; low vs high), serum uPA activity (categorical; low vs high), tumor cytoplasmic score (categorical; negative vs 1+ vs 2+) and nuclear score (negative vs positive). The variables were first evaluated with univariate analysis, and multivariate analysis was undertaken in a single step only for the factors showing significance of p < 0.1 on univariate analysis. A p-value of less than 0.05 was otherwise considered statistically significant. All statistical analyses were performed using a commercially available software program (SPSS Version 23.0, SPSS Inc., Chicago, IL).

## Results

### Serum uPA and suPAR quantification

The clinical characteristics of dogs included in serum analysis are summarized in **Table 1**. All 49 dogs received at least one dose of adjuvant carboplatin chemotherapy, but 11 dogs (22.4%) did not complete the intended 4 doses of chemotherapy due to metastatic disease development after limb amputation including 1 dog treated with 1 dose and 5 dogs each treated with 2 or 3 doses of carboplatin. Forty-five dogs (91.8%) were dead at the time of data collection and necropsy was performed in 17 dogs (34.7%) with histological confirmation of metastatic osteosarcoma as the causes of death. Four dogs (8.2%) were alive at the end of the study 679, 959, 1053 and 1413 days after diagnosis. No dogs were lost to follow-up. The median PFS and OS for the 49 dogs were 227 days [95% Confidence interval (CI) 123–331 days] and 277 days [95%CI 204–350 days], respectively.

**Table 1:** Characteristics of 49 dogs with osteosarcoma and 6 clinically healthy dogs used for serum uPA and uPAR analysis.

Characteristics	Discovery cohort	Validation cohort	Healthy
Dog number	26	23	6
Age (y)	8 (2–12)	8 (4–12)	4.5 (2–7)
Sex			
Spayed female	12 (46.2)	11 (47.8)	5 (83.3)
Castrated male	14 (53.8)	11 (47.8)	1 (16.7)
Intact male	0	1 (4.3)	0
Serum ALP activity			
Normal	21 (76.2)	21 (91.3)	6 (100)
High	5 (23.8)	2 (8.7)	0 (0)
Lymphocyte count (x10^9^ /L)	1.58 (0.79–5.25)	1.54 (0.45–3.05)	1.84 (0.26–2.42)
Monocyte count (x10^9^ /L)	0.48 (0–1.3)	0.48 (0.06–1.07)	0.16 (0.06–1.1)
Tumor location			
Scapula	1 (3.8)	1 (4.3)	Not applicable
Humerus	6 (23.1)	6 (26.1)	
Radius	12 (46.2)	6 (26.1)	
Ulna	1 (3.8)	1 (4.3)	
Femur	2 (7.7)	2 (8.7)	
Tibia	4 (15.4)	7 (30.4)	

Data are reported as median (range) for age, lymphocyte, and monocyte count, and number (%) of dogs in category for sex, serum ALP activity (relative to reference range), and tumor location.

**Fig 1:**
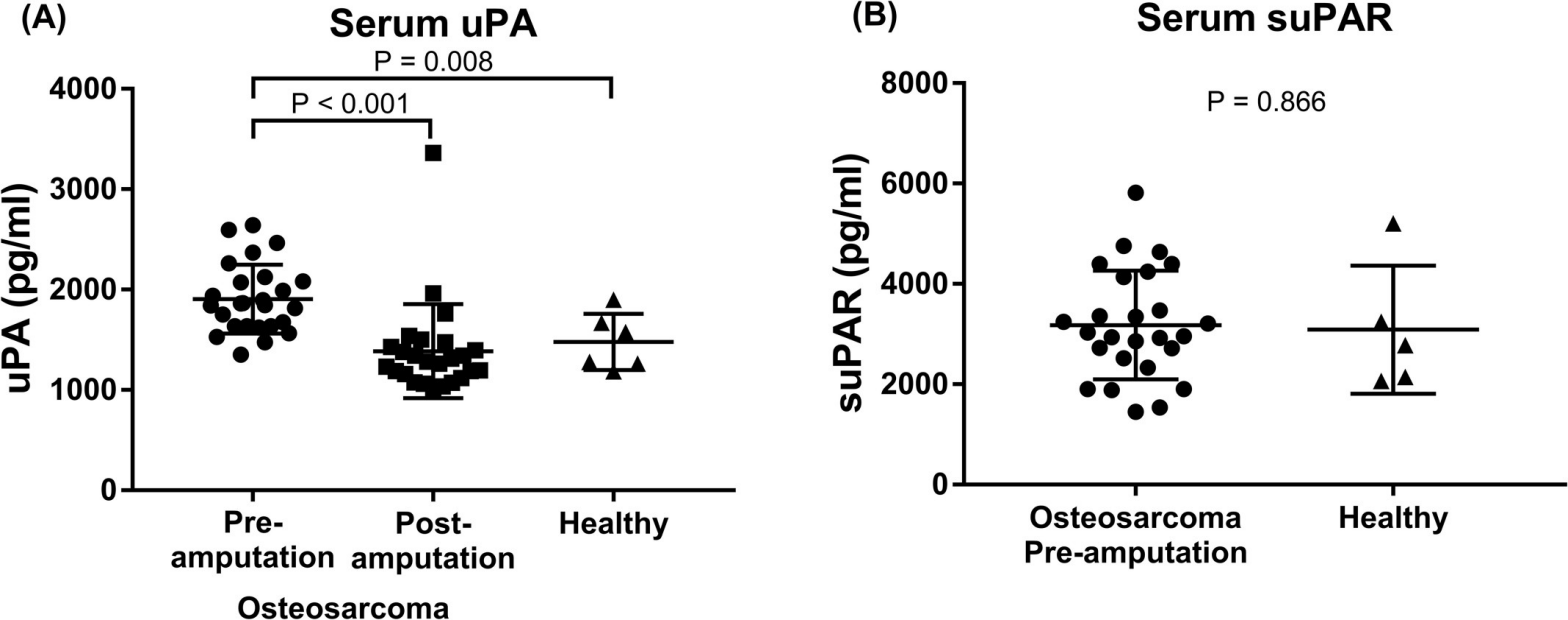
Serum uPA and suPAR levels of osteosarcoma-bearing and clinically healthy dogs. Serum uPA (A) and suPAR (B) levels were analyzed using commercially available canine uPA and uPAR ELISA kits, respectively. (A) Dogs with osteosarcoma had higher presurgical serum uPA level (n = 26) than clinically healthy dogs (n = 6; p = 0.008) and the level decreased postoperatively (n = 25; P < 0.001). (B) There was no difference in suPAR level between dogs with osteosarcoma pre-amputation (n = 26) and healthy control dogs (n = 5; p = 0.866). Serum suPAR level was not measured in the post-amputation samples, given the lack of difference between the osteosarcoma-bearing and healthy dogs at baseline. The error bars show standard deviation.

The serum uPA and suPAR levels were first compared between 26 dogs with osteosarcoma (discovery cohort) and 6 healthy dogs. The osteosarcoma-bearing dogs had significantly higher serum uPA at diagnosis (median 1905.4 pg/ml [95%CI 1767.2–2043.6 pg/ml]) than the clinically healthy control (median 1439.5 pg/ml [95%CI 1071.8–1807.3 pg/ml], p = 0.008; **Fig 1A**), while no difference was seen in suPAR level between the two groups (median 3179.4 pg/ml vs 3086.9 pg/ml, p = 0.866; **Fig 1B**). Analysis of the 25 paired pre- and post-amputation serum samples showed significant reduction in serum uPA postoperatively (median 1280.5 pg/ml [95%CI 1193.0–1580.2 pg/ml], p < 0.001; **Fig 1A**). Notably, there was no difference between the post-amputation and control groups (p = 0.268; **Fig 1A**). Serum suPAR level was not measured in the post-amputation samples, given the lack of difference between the osteosarcoma-bearing and healthy dogs at baseline. Only one dog had an increase in serum uPA postoperatively (from 2595.5 to 3360.4 pg/ml) at the time of carboplatin #1. The dog had a humeral osteosarcoma and pre-surgical staging tests with three view thoracic radiographs and abdominal ultrasound showed two 1–1.5 cm hepatic nodules without radiographically identifiable pulmonary nodules. Neither cytology or histology of the hepatic nodules was performed. This dog developed respiratory distress following carboplatin #2 and was euthanized due to radiographic evidence of pulmonary metastasis 68 days after limb amputation.

**Fig 2:**
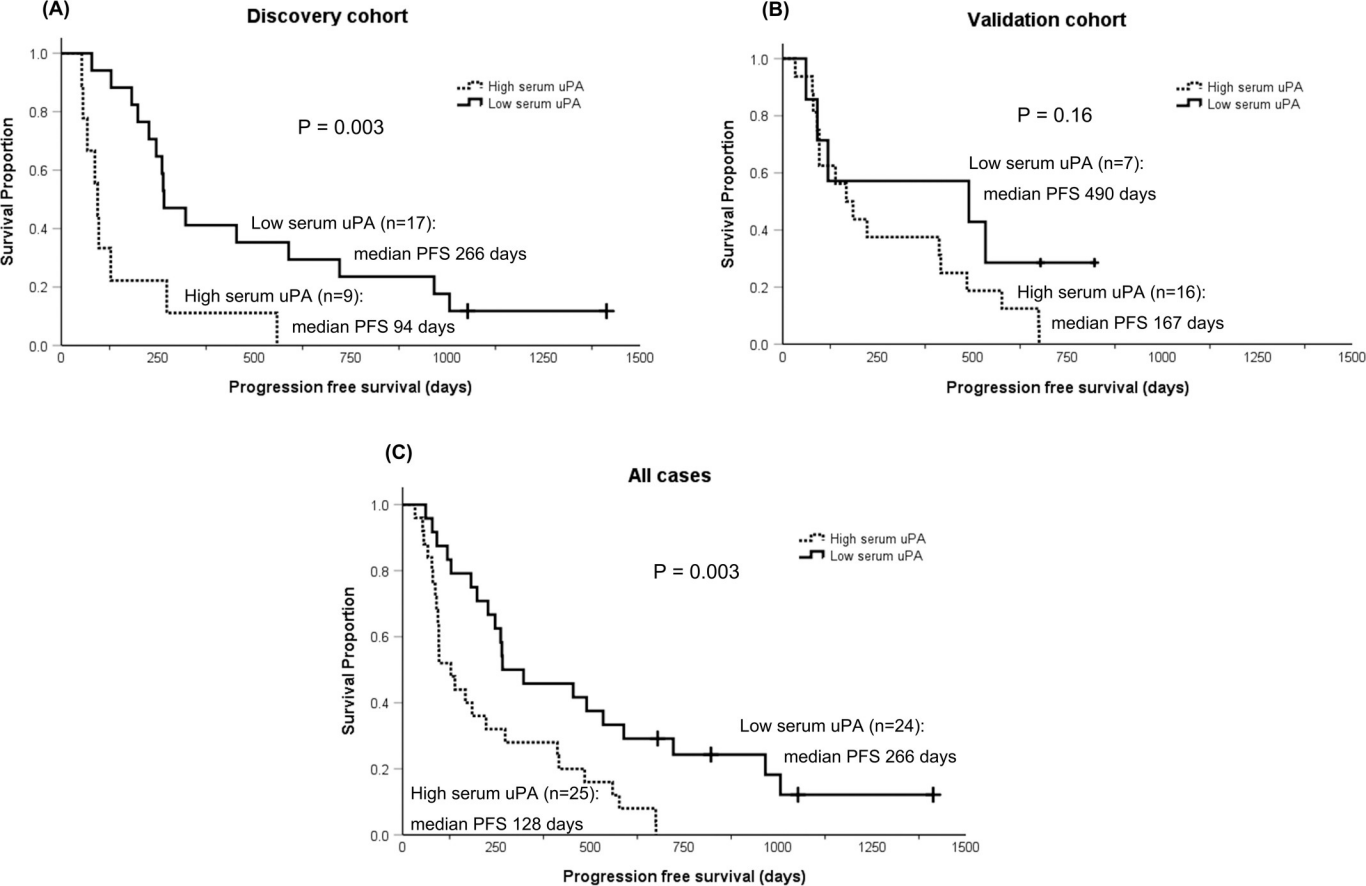
Kaplan-Meier curves for dogs with low or high serum uPA. Kaplan-Meier curves for progression free survival (PFS) of dogs with osteosarcoma treated with a limb amputation and carboplatin chemotherapy. (A) Kaplan-Meier curves for the 26 dogs in the discovery cohort. Median PFS was 266 days for dogs with low serum uPA (n = 17, solid line) versus 105 days for dogs with high serum uPA (n = 9, dashed line; p = 0.003). (B) Kaplan-Meier curves for the 23 dogs in the validation cohort. Median PFS was 490 days for dogs with low serum uPA (n = 7, solid line) versus 167 days for dogs with high serum uPA (n = 16; dashed line; p = 0.16). (C) Kaplan-Meier curves for all 49 dogs combining the discovery and validation cohorts. Median PFS was 266 days for dogs with low serum uPA (n = 24, solid line) versus 128 days for dogs with high serum uPA (n = 25, dashed line; p = 0.003).

For survival analysis, dogs were divided into two groups, high vs low (uPA/suPAR-H vs -L), based on cut-off values generated by X-Tile for each analysis in the discovery cohort. Dogs in the uPA-L group had significantly longer median PFS (266 days, p = 0.003) than dogs in the uPA-H group (median PFS 94 days; **Fig 2A** and **Table 2**). To validate the prognostic significance of serum uPA, serum samples of 23 dogs fulfilling the same criteria were analyzed (validation cohort). In this cohort, dogs in the uPA-L group had longer median PFS (490 days) than dogs in the uPA-H group (median PFS 167 days), but the difference was not statistically significant (p = 0.16; **Fig 2B**). However, in an analysis combining the both cohorts, significant difference was observed (median PFS; uPA-L 266 days vs uPA-H 128 days, p = 0.003) between the groups (**Fig 2C**). There was no difference in PFS or OS between dogs in the suPAR-L and the suPAR-H groups in both cohorts. These results are summarized in **Table 2**.

**Table 2:** Survival of osteosarcoma-bearing dogs with low or high serum uPA and suPAR levels.

	Progression Free Survival		Overall Survival	
	Median	95% CI	Median	95% CI
**uPA**				
Discovery cohort	**p = 0.003**		**p = 0.01**	
Low (n = 17)	266 days	184–348 days	338 days	124–551 days
High (n = 9)	94 days	74–114 days	123 days	70–176 days
Validation cohort	**p = 0.16**		**p = 0.436**	
Low (n = 7)	490 days	0–1442 days	534 days	0–1283 days
High (n = 16)	167 days	77–257 days	185 days	165–204 days
All dogs	**p = 0.003**		**p = 0.016**	
Low (n = 24)	266 days	34–498 days	338 days	51–625 days
High (n = 25)	128 days	58–198 days	180 days	118–242 days
**suPAR**				
Discovery cohort	**p = 0.216**		**p = 0.131**	
Low (n = 7)	198 days	0–465 days	238 days	0–579 days
High (n = 19)	261 days	196–326 days	319 days	168–470 days
Validation cohort	**p = 0.258**		**p = 0.123**	
Low (n = 16)	167 days	4–330 days	242 days	0–556 days
High (n = 7)	185 days	0–413 days	144 days	48–240 days
All dogs	**p = 0.648**		**p = 0.551**	
Low (n = 23)	198 days	68–328 days	238 days	113–363 days
High (n = 26)	246 days	136–336 days	286 days	195–377 days

### Serum uPA activity

**Fig 3:**
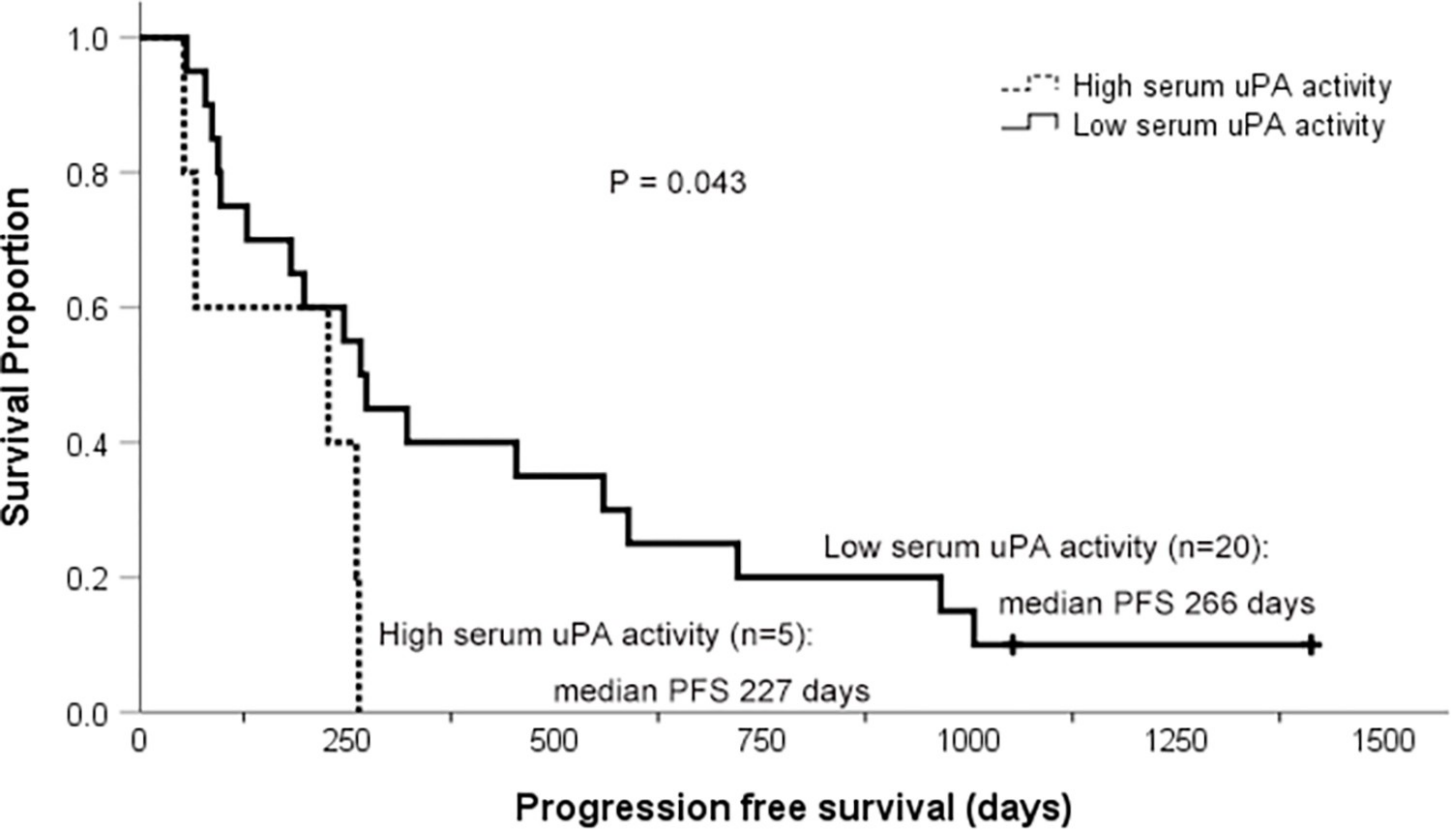
Kaplan-Meier curves for dogs with low or high serum uPA activity. Kaplan-Meier curves for progression free survival (PFS) of dogs with osteosarcoma treated with a limb amputation and carboplatin chemotherapy. Median PFS was 266 days for dogs with low serum uPA activity (n = 20, solid line) and 227 days for dogs with high serum uPA activity (n = 5, dashed line; p = 0.043).

Serum uPA activity (uPAact) level of the 26 dogs with osteosarcoma in the discovery cohort and the 6 heathy dogs was evaluated. One sample from each group was excluded due to hemolysis. There was no difference in uPA activity between dogs with osteosarcoma and the control group (median 2001 IU/μl vs 2057 IU/μl, p = 0.734). Within the osteosarcoma population, dogs were divided into two groups, high vs low (uPAact-H vs -L), and dogs in the uPAact-L group had significantly longer median PFS (266 days) than dogs in the uPAact-H group (227 days, p = 0.043; **Fig 3**). The median OS was also longer in the uPAact-L group (319 days) than the uPAact-H group (261 days), but the difference was not statistically significant (p = 0.071). These results are summarized in **Table 3**.

**Table 3:** Survival times of osteosarcoma-bearing dogs with low or high serum uPA activity.

	Progression Free Survival		Overall Survival	
	Median	95% CI	Median	95% CI
uPA activity	p = 0.043		p = 0.071	
Low (n = 20)	266 days	207–325 days	319 days	126–512 days
High (n = 5)	227 days	0–570 days	261 days	0–557 days

### Tissue and cell line uPA and uPAR analysis

**Fig 4:**
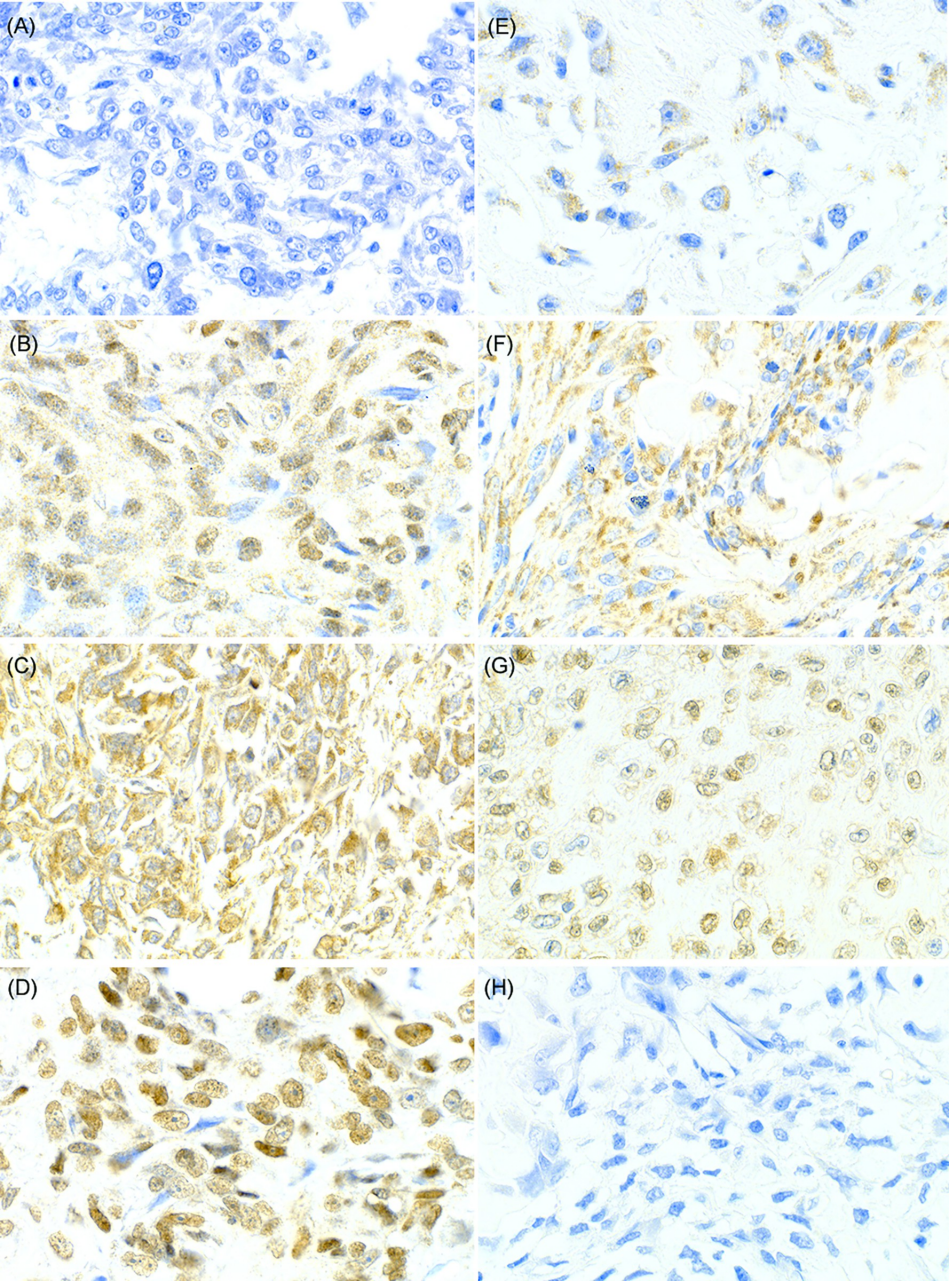
Representative tissue microarray cores with uPA and uPAR immunohistochemical labeling. (A) A core with negative uPA/uPAR expression. Osteosarcoma cells had clear cytoplasm and nuclei with no immunoreactivity. (B) Diffuse and weak cytoplasmic uPA expression (1+) is seen in majority of the osteosarcoma cells with nuclear positivity. (C) Osteosarcoma cells diffusely showed strong cytoplasmic uPA expression (2+) throughout the core with nuclear positivity. (D) Strong distinct nuclear uPA expression is present with weak cytoplasmic labeling (1+). (E) Weak to moderate uPAR expression (1+) is present in cytoplasm of the osteosarcoma cells. (F) Cytoplasmic uPAR expression is strong (2+) and seen diffusely in this core sample. (G) Nuclei of most osteosarcoma cells are positive for uPAR expression with weak cytoplasmic positivity (1+). (H) Negative control with primary antibody omission. Images taken at 400x.

**Fig 5:**
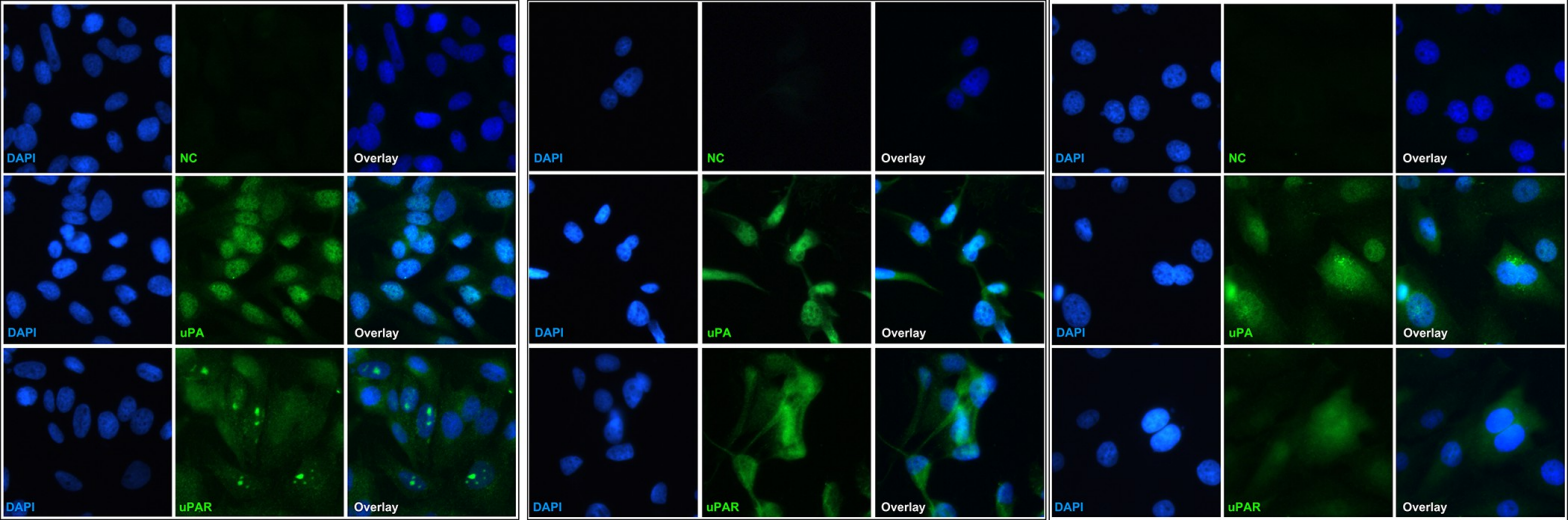
Immunofluorescence images of uPA and uPAR expression in osteosarcoma cell lines. Both diffuse cytoplasmic and nuclear uPA and uPAR positivity are seen in one human osteosarcoma cell line (A) and two canine cell lines D17 (B) and Dharma (C). Nuclear expression was strong in uPA and weak to moderate in uPAR in all three cell lines. Nuclei were stained with DAPI (blue). Primary antibody was omitted in negative control (NC). Images taken at 400x.

Among the 58 osteosarcoma tumors evaluated, the majority expressed uPA (n = 44, 75.9%) and/or uPAR (n = 45, 77.6%) diffusely within the cytoplasm (**Fig 4**). Strong (2+) cytoplasmic uPA intensity was seen in 6 tumors (10.3%) while the majority of uPA cytoplasmic expression was weak to moderate (n = 38, 65.5%). Similarly, strong cytoplasmic uPAR expression was seen in 16 cases (27.6%). Nuclear uPA and uPAR expression was also noted in 16 (27.6%) and 2 (3.4%) tumors, respectively (**Fig 4**). All tumors with nuclear immunoreactivity also had cytoplasmic labeling for the corresponding proteins. Immunofluorescence using the same antibodies confirmed diffuse cytoplasmic and nuclear uPA and uPAR expression in all three canine osteosarcoma cell lines as well (**Fig 5**). While its significance is unknown, aggregated nuclear uPAR labeling was seen in HOS cell line (**Fig 5A**).

Forty-one (70.7%) of 58 tumors including both primary and metastatic sites co-expressed uPA and uPAR in the cytoplasm, while both were negative in 10 tumors (17.2%). McNemar’s test showed a significant association between uPA and uPAR cytoplasmic positivity (p < 0.001), suggesting the presence of autocrine and paracrine activation of the uPA system in canine osteosarcoma.

Expression patterns were compared between the 34 primary and 24 metastatic tumors from 38 dogs. Paired primary bone and metastatic lymph node samples were available for 2 dogs from limb amputation. The remaining 22 metastatic samples were from necropsy of 8 dogs, among which primary bone samples from limb amputation at diagnosis were also available in 5 dogs. The interval between limb amputation and necropsy of these 5 dogs were 87, 94, 259, 295, and 753 days. The majority of primary tumors had cytoplasmic uPA (91.4%) and/or uPAR (94.1%) expression, while approximately half of the metastatic tumors expressed cytoplasmic uPA (54.2%) and/or uPAR (54.2%) **(Table 4)**. Both primary and metastatic tumor tissues were concurrently available only in 5 dogs, and therefore no statistical analysis was performed on these samples.

**Table 4:** uPA and uPAR positivity in primary and metastatic osteosarcoma.

	Primary (n = 34)	Metastasis (n = 24)
**Cytoplasmic uPA positive**	31 (91.2%)	13 (54.2%)
**Nuclear uPA positive**	15 (44.1%)	1 (4.2%)
**Cytoplasmic uPAR positive**	32 (94.1%)	13 (54.2%)
**Nuclear uPAR positive**	2 (5.9%)	0 (0%)

Prognostic significance of tumor uPA and uPAR expression was analyzed in 28 dogs that underwent SOC, of which clinical characteristics are summarized in **Table 5**. Eight dogs (28.6%) did not complete the intended 4 doses of carboplatin protocol due to development of metastatic disease including 2 dogs treated with 1 dose, 4 dogs with 2 doses, and 2 dogs with 3 doses of carboplatin. Twenty-seven dogs (96.4%) were dead while one dog was alive at the time of data collection (1413 days after amputation). No dogs were lost to follow-up. The median PFS and OS for the 28 dogs were 198 days [95%CI 158–238 days] and 286 days [95%CI 167–405 days], respectively. Neither tumor uPA nor uPAR expression was associated with either PFS or OS (**Table 6**).

**Table 5:** Characteristics of 28 dogs with osteosarcoma used for tumor uPA and uPAR analysis.

Characteristics	
Age (y)	8 (2–12)
Sex	
Spayed female	15 (53.6)
Castrated male	12 (42.9)
Intact male	1 (3.6)
Serum ALP activity	
Normal	25 (92.6)
High	2 (7.4)
Lymphocyte count (x10^9^ /L)	1.60 (0.6–5.25)
Monocyte count (x10^9^ /L)	0.46 (0–1.3)
Tumor location	
Scapula	1 (3.5)
Humerus	7 (25)
Radius	11 (39.3)
Femur	5 (17.9)
Tibia	4 (14.3)

Data are reported as median (range) for age, lymphocyte, and monocyte count, and number (%) of dogs in category for sex, serum ALP activity (relative to reference range), and tumor location.

**Table 6:** Survival times of 28 osteosarcoma-bearing dogs with different uPA and uPAR tumor expression.

	Progression Free Survival		Overall Survival	
	Median	95% CI	Median	95% CI
Cytoplasmic uPA	p = 0.893		p = 0.865	
Negative (n = 3)	179 days	48–310 days	279 days	140–418 days
1+ (n = 20)	208 days	182–234 days	295 days	142–4481 days
2+ (n = 5)	175 days	0–431 days	264 days	0–693 days
**Nuclear uPA**	**p = 0.406**		**p = 0.281**	
Negative (n = 15)	198 days	15–381 days	286 days	103–469 days
Positive (n = 13)	208 days	167–249 days	295 days	121–469 days
**Cytoplasmic uPAR**	**p = 0.861**		**p = 0.836**	
Negative (n = 1)	480 days	NA	480 days	NA
1+ (n = 18)	179 days	135–223 days	279 days	179–379 days
2+ (n = 9)	264 days	106–422 days	295 days	204–386 days
**Nuclear uPAR**	**p = 0.374**		**p = 0.928**	
Negative (n = 26)	198 days	159–237 days	295 days	165–425 days
Positive (n = 2)	63 days	NA	166 days	NA

### Prognostic factor and correlation analysis

Cox regression analysis of 6 purported prognostic factors and the measured uPA and uPAR levels in the current study was performed in the 25 dogs with appendicular osteosarcoma that underwent SOC and serum samples were available. Univariate analysis showed tumor location, serum ALP status, peripheral monocyte count, serum uPA, and serum uPA activity as risk factors (p < 0.1) for shorter PFS. Of those, peripheral monocyte count (p = 0.049), serum uPA (p = 0.003), and serum uPA activity (p = 0.006) remained significant on multivariate analysis. For OS analysis, tumor location, serum ALP status, high peripheral lymphocyte count, serum uPA, and serum uPA activity resulted in a p-value of < 0.1 on univariate analysis, but multivariate analysis identified only serum ALP status (p = 0.024), high serum uPA (p = 0.008), and high serum uPA activity (p = 0.026) as risk factors. These results are summarized in **Table 7**.

**Table 7:** Risk factors in 25 osteosarcoma-bearing dogs with p-values and hazard ratios in multivariate analysis.

Progression Free Survival		
	p-value	Hazard Ratio [95% CI]
Location	0.085	3.67 [0.84–16.09]
(humerus vs others)		
Serum ALP	0.051	5.09 [0.99–26.14]
(abnormal vs normal)		
Monocyte count	**0.049**	2.58 [1.01–6.60]
(high vs low)		
Serum uPA	**0.003**	5.54 [1.81–16.93]
(high vs low)		
Serum uPA activity	**0.006**	7.72 [1.78–33.57]
(high vs low)		
**Overall Survival**		
Location	0.285	2.09 [0.54–8.04]
(humerus vs others)		
Serum ALP	**0.024**	6.09 [1.26–29.32]
(abnormal vs normal)		
Lymphocyte count	0.745	0.84 [0.28–2.48]
(high vs low)		
Serum uPA	**0.008**	4.75 [1.50–15.01]
(high vs low)		
Serum uPA activity	**0.026**	5.40 [1.22–23.87]
(high vs low)		

**Fig 6:**
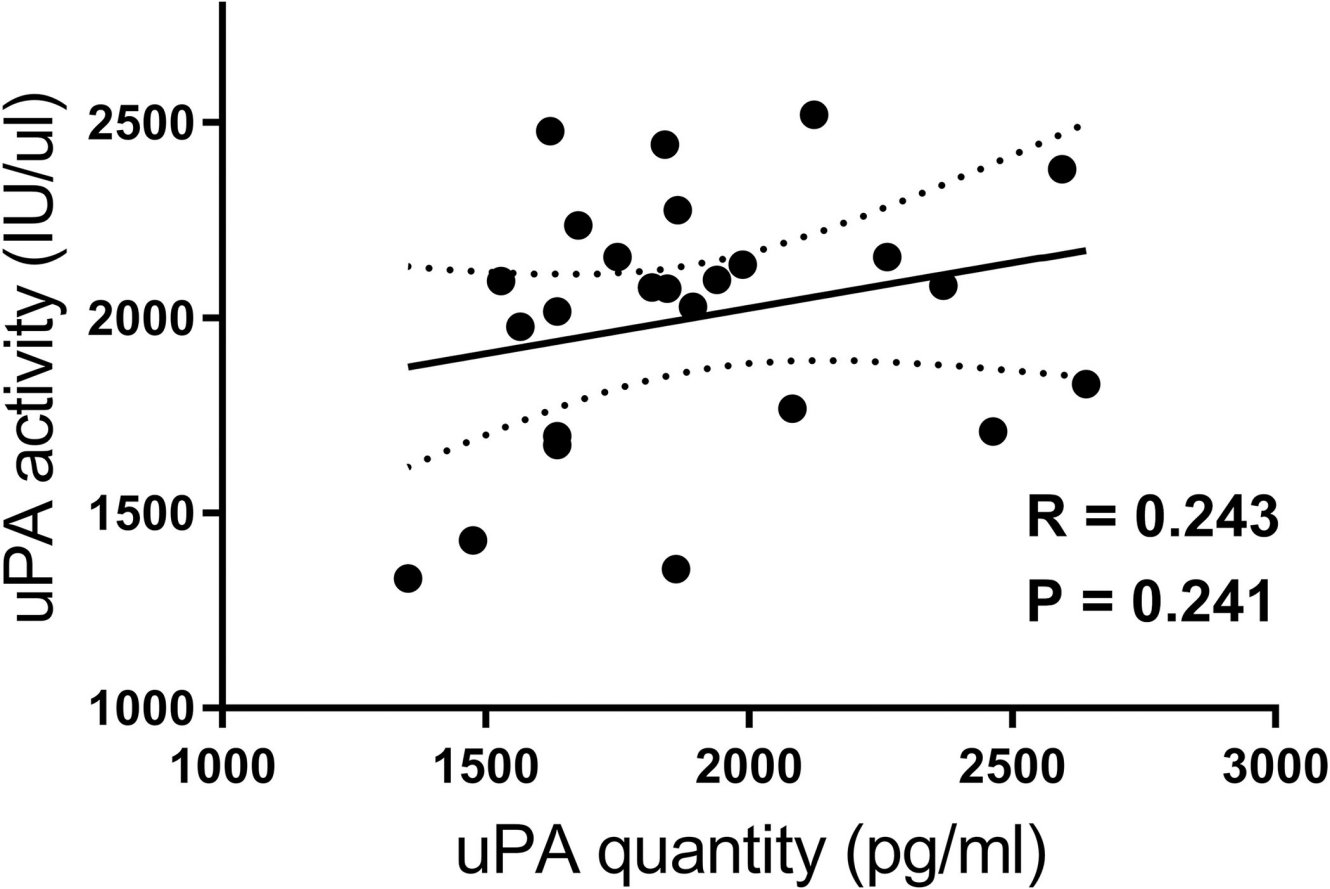
Pearson’s correlation between serum uPA quantity and activity in 25 dogs with osteosarcoma. The paired serum uPA quantity measured by canine uPA ELISA kit and uPA activity measured by a chromogenic uPA activity kit in 25 dogs with osteosarcoma were plotted for correlation analysis. A Pearson’s correlation coefficient showed poor correlation between serum uPA quantity and activity (p = 0.241, R = 0.241). The dotted lines represent 95%CI.

Correlation analysis showed poor correlation between serum uPA level and uPA activity (r = 0.243, p = 0.241; **Fig 6**). Within the population where both serum ELISA and tissue immunohistochemistry analysis were performed (n = 13), all tissue samples expressed cytoplasmic uPA at intensity of either 1+ (n = 12) or 2+ (n = 1) and uPAR at 1+ (n = 8) or 2+ (n = 5). When the serum suPAR level was compared between dogs with the two tumoral uPAR cytoplasmic intensities, there was no statistically significant difference (p = 0.88). Due to the skewness of the data in the population, serum uPA level was not compared according to the tumor tissue uPA immunoreactivity.

## Discussion

The uPA system consists of the ligand uPA and its receptor uPAR as well as their inhibitors PAI-1 and PAI-2. Binding of uPA to uPAR results in conversion of pro-uPA to uPA, then subsequently localizes the proteolytic uPA activity to the leading edge of the tumor and initiates uPAR interaction with other receptors, thus both uPA and uPAR contributes to cancer metastasis, invasion, and angiogenesis [8, 31, 32]. One of the significant findings of the current study was the high prevalence (>80%) of uPA and uPAR expression in canine osteosarcoma tumors with concurrent detection in the systemic circulation of the affected dogs. About three quarters of the TMA cores expressed both proteins concurrently, suggesting autocrine/paracrine activation of the system within the same tissue or possibly cells. Furthermore, the significant postoperative reduction in circulating uPA levels supports the hypothesis that tumor tissue is the primary source of uPA production. The released proteins could further contribute to progression and development of metastasis systemically, and could therefore represent a potential treatment target. One example of emerging uPA/uPAR targeted therapy is eBAT, a bispecific toxin targeting uPAR and EGFR [16]. A phase I/II trial of dogs with splenic hemangiosarcoma reported encouraging results with improved median survival time (8.1 months vs 4.9 months) and 6-month survival rate (65.2% vs 38.7%) in dogs treated with adjuvant eBAT followed by doxorubicin compared to the historical control group that received adjuvant doxorubicin alone [16]. Combined with the reported up-regulation of EGFR gene in canine osteosarcoma, a similar therapeutic approach may therefore be effective for canine osteosarcoma as well [16].

Another intriguing finding of the current study is the prognostic significance of serum uPA quantity and activity (**Figs 2** and **3**). Dogs with high serum uPA quantity and activity had significantly shorter survival (**Tables 2** and **3**). This significance remained following multivariate analysis of the entire population (**Table 7**) even when analyzed with known prognostic factors of canine osteosarcoma such as serum ALP, tumor location, and peripheral monocyte/lymphocyte count [25–27]. In dogs, the only study measuring serum uPA compared the levels between clinically-healthy and cancer-bearing dogs [33]. No difference was noted between the groups, but the lack of difference is likely due to the wide variety of different tumor types included. One direct consequence of increased uPA production and activation is increased conversion of plasminogen to plasmin, which degrades extracellular matrix proteins directly, but also indirectly via activation of matrix metalloproteinases and release of growth factors [8]. The presence of active plasmin is critical in cancer metastasis development as shown by plasminogen deficiency resulting in inhibition of pulmonary and nodal metastasis without affecting main tumor growth [34]. As an activator of pro-uPA, uPAR binds to pro-uPA as well as the active uPA fragment and subsequently may enhance plasminogen conversion both locally at the invasive tumor front with uPAR and systemically with suPAR binding [35, 36]. Therefore, an increase in serum uPA is likely a reflection of the malignant biological features of a cancer and/or large burden of invasive and metastatic neoplasia as well as a reflection of the tumor microenvironment. As such, serial measurements of serum uPA in osteosarcoma patients may also reveal correlation between circulating uPA levels and disease progression. The only dog that had elevation in serum uPA postoperatively in the current study had the second shortest survival time due to pulmonary metastasis. While the dog was not symptomatic for respiratory disease and no thoracic imaging was performed at the time of second blood collection, the dog may have had detectable pulmonary nodules. Nevertheless, these findings are sufficient to encourage prospective analysis of serum uPA in a larger cohort of cases at risk for metastasis.

While the serum uPA analysis showed significance, two major questions that remain are the biological reasons why the uPA quantity and activity did not correlate to each other and why suPAR was not prognostic or elevated in osteosarcoma bearing dogs. The former may be explained by the biological complexity of the uPA molecule. Enzymatically non-functional pre-uPA can be cleaved to high molecular weight uPA then to low molecular weight (LMW) uPA after release of inactive amino terminal fragment (ATF) by plasmin [37]. While all uPA forms including ATF can bind to uPAR, only LMW uPA has catalytic activity and converts plasminogen to plasmin [37]. The chromogenic uPA activity kit used in the current study quantifies uPA activity based on plasminogen catalysis, hence reflects the amount of the LMW uPA form exclusively. The antibody used in the uPA ELISA kit was not commercially available separately but full-length canine uPA was reportedly used as the immunogen of this polyclonal antibody. Thus, we speculate that the measured serum uPA quantity includes one or multiple uPA forms present in serum, resulting in a discrepancy between the values. Another aspect of the discrepancy in uPA activity and quantity levels is the presence of uPA inhibitor PAI-1/2, which can inhibit plasminogen conversion by uPA. Overall, the fact that both uPA quantity and activity were prognostic strongly supports that uPA contributes to tumor malignancy possibly both in uPAR-dependent and independent manners.

While serum suPAR was not elevated or prognostic in dogs with osteosarcoma, the overproduction of uPA would still activate the cellular uPAR molecules subsequent to their interaction and facilitate tumor invasion/metastasis. Thus, the lack of increased serum suPAR level in dogs with osteosarcoma may rather indicate poor solubilization of uPAR in this disease setting. The receptor solubilization is catalyzed by multiple enzymes such as phospholipases or chemotrypsins and the released suPAR form may act as an endocrine messenger that induces uPAR-induced signaling even in cells without uPAR expression [38, 39]. Therefore, increased uPAR shedding would further activate the uPA system but would not be critical for tumor malignancy. Local or systemic catalytic enzyme activities were not evaluated in our cohorts, and therefore it was not possible to investigate these possibilities. Another possible cause of the lack of suPAR elevation or prognostication is a lack of measurement of specific suPAR forms. Upon solubilization, suPAR can be present as one of three different forms depending on the cleavage sites [24]. Similar to uPA, the antibody from the uPAR ELISA kit was not available but full-length uPAR was reportedly used as the immunogen. Thus, it is possible that certain suPAR forms were released in significant amounts but were not detected by the assay.

This report is the first study showing nuclear localization of uPA and uPAR in osteosarcoma. Distinct nuclear expression was evident in about half of the tumors for uPA and uncommonly for uPAR (**Table 6**). The immunofluorescent labeling pattern in canine osteosarcoma cell lines was consistent with the pattern reported in human cell lines [10]. Once these proteins are produced intracellularly, uPA is shed to the extracellular space while uPAR is localized to the cellular membrane to function as a receptor. Degraded uPAR can be endocytosed by low-density lipoprotein-related receptor (LRP) to recycle or redistribute, and therefore, both uPA and uPAR are predominantly located within the cytoplasm. In addition, pre-uPA, uPAR, and the uPA/uPAR complex bind to nucleolin and are translocated to the nucleus, then regulate transcription of genes, such as VEGFR1 and VEGFR2 [10–12]. uPA/uPAR nuclear translocation may therefore indicate more biologically aggressive features of the tumor. Indeed nuclear positivity has been documented immunohistochemically in human breast cancer and squamous cell carcinoma as well as canine hemangiosarcoma tissues, yet their clinical significance is to be determined [40–42]. In a report by Jankun et al., where uPA and uPAR nuclear positivity was observed “sometimes” and “occasionally” respectively, they were only present in malignant but not benign breast cancer tissues, but no prognostic analysis was performed [40].

One limitation of the current study is lack of specificity testing of the antibodies used. Two commercially available clinically-used human uPA and uPAR ELISA kits were also tested but lack of reactivity with canine proteins was confirmed. The antibodies in the canine specific kits used were not available and their specificity could not be determined by other methods such as western blotting using the serum or canine tissue/cell line samples. Also, the prognostic significance of uPA and uPAR needs to be carefully interpreted as the data is only pertaining to the certain population of dogs that presented with appendicular osteosarcoma without gross metastatic disease and underwent standard therapy. The limited but uniform patient selection yielded good clinically follow-up with less than 10% censoring rate and consists of standardized treatments that are clinically applicable. Another limitation is the retrospective nature of the study cohort. Case selection bias is not present between the groups analyzed, since both tissue and serum uPA and uPAR data was obtained later and not provided to the clinicians. However, it is possible that some cases had gross metastatic disease that was not detected by the baseline staging tests, that included three view thoracic radiographs in all cases but not all for abdominal ultrasound and thoracic/abdominal CT scan, and therefore, some of the elevated baseline serum uPA/uPAR levels may not accurately represent the profiles of dogs with appendicular osteosarcoma without gross metastatic disease.

## Conclusion

In conclusion, our results showed high utilization of the uPA pathway and association with disease progression in canine osteosarcoma. Although their prognostic significance should be validated prospectively, the high prevalence of tumor uPA and uPAR expression may suggest that the uPA system may serve as a potential therapeutic target in canine osteosarcoma.
